# Assessment of Possible Application of an Atmospheric Pressure Plasma Jet for Shelf Life Extension of Fresh-Cut Salad

**DOI:** 10.3390/foods10030513

**Published:** 2021-03-01

**Authors:** Tiziana Silvetti, Matteo Pedroni, Milena Brasca, Espedito Vassallo, Giacomo Cocetta, Antonio Ferrante, Ivano De Noni, Laura Piazza, Stefano Morandi

**Affiliations:** 1 Department of Food, Environmental and Nutritional Sciences, Università degli Studi di Milano, Via G. Celoria 2, 20133 Milan, Italy; tiziana.silvetti@ispa.cnr.it (T.S.); ivano.denoni@unimi.it (I.D.N.); 2 National Research Council, Institute of Sciences of Food Production, Via G. Celoria 2, 20133 Milan, Italy; stefano.morandi@ispa.cnr.it; 3 National Research Council, Institute for Plasma Science and Technology, Via R. Cozzi 53, 20125 Milan, Italy; matteo.pedroni@istp.cnr.it (M.P.); espedito.vassallo@istp.cnr.it (E.V.); 4 Department of Agricultural and Environmental Sciences, Università degli Studi di Milano, Via G. Celoria 2, 20133 Milan, Italy; giacomo.cocetta@unimi.it (G.C.); antonio.ferrante@unimi.it (A.F.); 5 Department of Environmental Science and Policy, Università degli Studi di Milano, Via G. Celoria 2, 20131 Milan, Italy; laura.piazza@unimi.it

**Keywords:** atmospheric pressure cold plasma, antimicrobial, ready-to-eat salad, minimally processed, shelf life

## Abstract

Ready-to-eat salads are very perishable with quality losses within 6–7 days, and the extension of their shelf life is still a challenge. In this work, an atmospheric pressure plasma jet (APPJ) was applied for the surface decontamination of fresh-cut lettuce baby leaves. The APPJ antimicrobial efficiency on the natural microbiota and its impact on some physicochemical attributes of lettuce were evaluated as a function of the treatment duration (0–30 s). Then, the influence of plasma treatment on the salad shelf life was studied, following the growth of aerobic mesophilic bacteria in both untreated and plasma-treated samples during 9 days of storage at 4 °C, together with the plasma-induced changes in physicochemical parameters of lettuce leaves. The APPJ induced a fast (15 s) microbial decontamination (1.3 log_10_ CFU/g) of the salad surface. Exposure time and salad-plasma plume distance were the parameters that substantially affected the microbial inactivation. APPJ treatment retarded bacterial growth during the refrigerated storage, as plasma-treated samples were noticeably less contaminated than the non-treated ones in the first 3–4 days. No significant effect were observed on electrolyte leakage, pH, and dry matter content in both the set up phase and the shelf life study.

## 1. Introduction

Fresh-cut vegetables and fruits are increasingly consumed owing to their nutritional benefits. Moreover, they meet the consumer demand for ready-to-eat food requiring minimal effort and time for preparation [1]. Nevertheless, a significant part of fresh produce (20–40%) undergoes microbial spoilage, which influences quality, safety, and shelf life [2]. It has to be stressed that it is essential to start with a fresh raw material of high quality in order to offer a ready-to-eat product of high hygienic quality. The microbial load may be different in leafy vegetables grown in open field, in greenhouse, or in hydroponic soilless systems. Usually, soilless systems are more effective in controlling microbial contamination, both at harvest and during storage [3]. Thus, the safety and spoilage issues of fresh produce are still of interest even though important advances have also been made in postharvest packaging and refrigeration strategies [2,4]. Besides, washing has an economic and environmental impact owing to the large amount of water necessary to provide an adequate water quality for its intended use. One must keep in mind that minimization of water consumption and wastewater discharge rates is really one challenge for the food industry. Therefore, it is of paramount importance to preserve water quality throughout the entire washing process, i.e., maintaining it at a reasonable bacteriological quality, as water could serve as a source of cross-contamination. Thus, the use of an efficient decontamination technique that produces a limited amount of wastewater per unit mass of product would allow a high recycling ratio and, thereby, would be an environment-friendly alternative [5]. In addition, optimized postharvest decontamination and sanitation treatments of fresh minimally processed products involve chlorine washing, but the formation of potentially carcinogenic chlorinated organic compounds and the limited efficiency in reducing foodborne pathogens at the concentrations normally used (50–200 ppm) stimulate the development of alternative and “green” methods to ensure food safety [6,7]. There is especially great interest in new (non-thermal) antimicrobial technologies and, in recent years, atmospheric pressure cold plasma has attracted much attention as a promising unconventional processing. This process has some benefits, such as the ability to provide a vast array of chemically active species without extreme process conditions and expensive vacuum equipment. However, there are several methods of plasma generation and a wide range of possible geometries. Each type of plasma source has both advantages and limitations, along with high variation of the antimicrobial efficiency [8]. For example, as reported from Baier et al. [9], a dielectric barrier discharge (DBD) is often used in many studies of fruit and vegetable decontamination, allowing a uniform exposure of the whole sample. Nevertheless, this technique showed significant impacts on the salad leaf tissue. Atmospheric plasma jet was revealed to be a more suitable plasma source for such susceptible samples. In fact, the limited penetration depth of the reactive plasma species enables an effective superficial chemical action, without affecting the nutrients and the quality of the treated food [10]. However, a basic requirement is to avoid plasma instability such as the glow-to-arc transition [11]. In fact, local arc formation leads to safety problems as well as commercially unacceptable damages of the plasma-treated food. The application of a high-voltage nanopulses generator as an energy source allows to use only compressed air to form the plasma without the addition of noble gases (Ar and He). The operation with ambient air strongly reduced the system complexity and operation costs. In this work, a homemade atmospheric pressure plasma jet (APPJ) [12] was evaluated as a decontamination tool for green leaf lettuce (*Lactuca sativa*). To this aim, a preliminary trial was performed to define the optimum process parameters required for successful sanitization of unwashed, naturally contaminated lettuce leaves, without interfering with the physiological properties of the plant. The outcomes were subsequently used in a shelf life study. We focused on the inherent microbiota of salad, without using intentionally inoculated samples. Actually, previous studies highlighted the importance of considering the naturally occurring microbiota residing on leafy vegetables, as it may be more and thus persistent than potential human pathogens. The native microbiota showed higher resilience to environmental conditions as compared with the inoculated pathogenic strain [13]. Moreover, the leaf-associated population is diverse, so consumers are exposed to about 50 bacterial species during salad consumption. In particular, lettuce leaves often harbour high populations of non-pathogenic bacteria [14,15]. This community consists of physiologically different species that might possess a different resistance to cold plasma. Hence, our approach aimed to mimic the risk assessment under the worst foreseeable conditions of use, i.e., in the presence of the indigenous microbiota.

## 2. Materials and Methods

### 2.1. Plant Material

The experiment was conducted on green baby lettuce (*Lactuca sativa* L., var. acephala) supplied by Bonduelle Italia srl (San Paolo d’Argon, Bergamo, Italy). Baby lettuce leaves were harvested at the time they had a length of 5–10 cm. The unprocessed (prior to sorting and washing operations) lettuce was provided to the laboratory in polypropylene bags without use of modified atmosphere, in the form of a standard sample (300–400 g) used by Bonduelle for physico-chemical quality evaluation. The transport from the company to the laboratory was carried out using a refrigerated box to ensure appropriate and constant temperature, without interrupting the cold chain.

### 2.2. Plasma Device

The device used for the treatments was an APPJ developed at the Institute for Plasma Science and Technology (ISTP-CNR) and previously described by Pedroni et al. [12]. The APPJ consists of two coaxial electrodes (1 mm electrodes distance) between which air flows at a high rate (5–20 L/min). The device is powered by a pulsed power generator (FID GmbH—FPG 30-100MC4S10) via coaxial high-voltage cables. It delivers nanosecond voltage square pulses up to 30 kV at repetition rates from 12 to 100 kHz. The pulses’ rise time is 2–3 ns and their width at 90% is 13–15 ns. The supplied voltage is measured by a 1/1000 voltage probe (Tektronix P6015A, 75 MHz), and the electrical signals are visualized by means of a Tektronix digital oscilloscope (500 MHz). The APPJ is installed in a Faraday cage in order to avoid electromagnetic interferences. The dry air flow was adjusted using a flowmeter.

### 2.3. Salad Treatment and Storage Conditions

**Figure 1:**
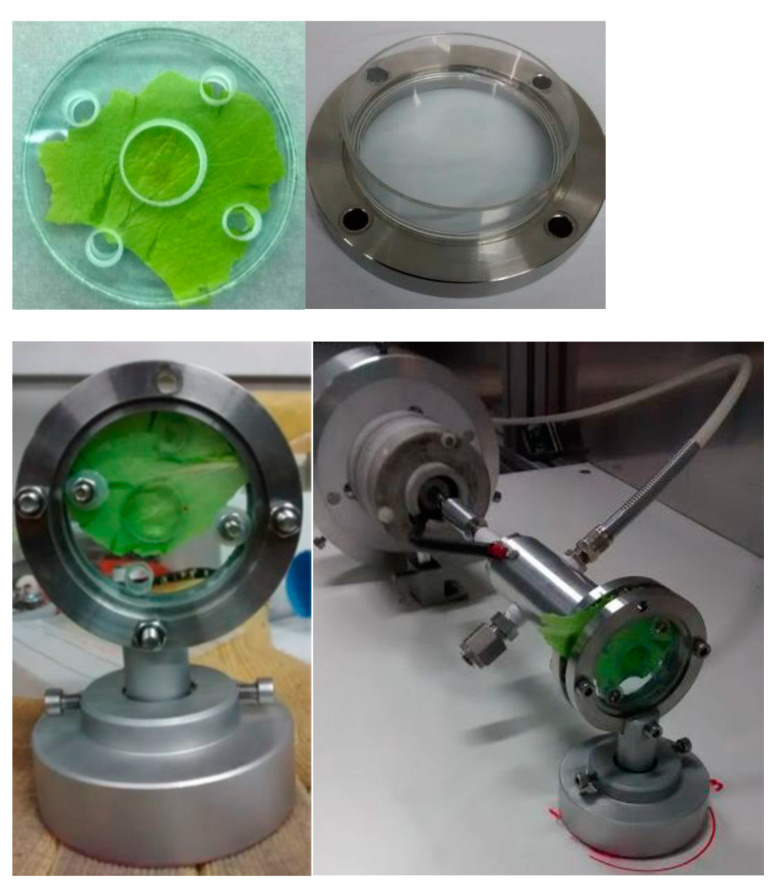
Experimental set up.

For each treatment, six leaves were randomly selected from each sample (300–400 g) of unprocessed lettuce. Of these, three were subjected to the treatment, while the other three represented the control. Every lettuce leaf was treated alone, by placing it on a glass support fixed in a sample holder metal cage, which can be positioned at different distances in front of the APPJ (Figure 1).

**Figure 2:**
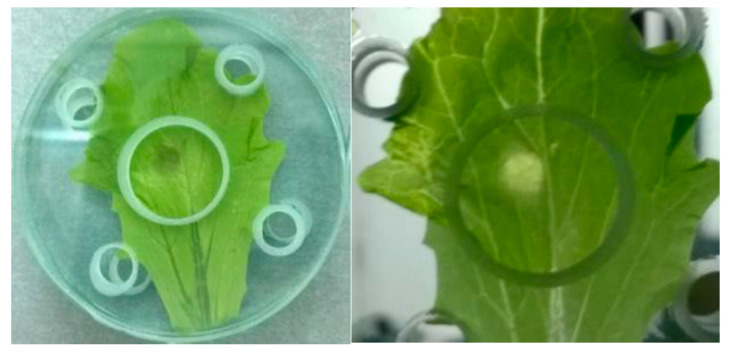
Example of visible plasma-induced damage.

The sample holder geometry was designed to ensure correct sample placement and avoid leaf removal due to the gas outflow. Moreover, single leaves can be treated on both faces by turning them 180 degrees. The central hole is 2 cm^2^, which corresponds to the maximum area effectively covered by the plasma plume [12]. This work required a preliminary experimental step for equipment set up in order to determine the adequate operating conditions expressly for salad leaves (i.e., exposure time), which were applied in a subsequent step concerning shelf life evaluation. The treatments were carried out with plasma parameters already optimized by Pedroni et al. [12]. They were 7.5 kV of applied voltage, 20 kHz of pulses’ frequency, and 20 L/min of air flow. These conditions led to the highest production of plasma reactive species with bactericidal activity. Regarding the salad-plasma plume distance, this was evaluated by visual analysis of leaves treated at different distances (1 to 4 cm) from the APPJ for 60 s. Long treatments led to the appearance of unacceptable damages of the plasma-treated lettuce surface. An example of this kind of plasma-induced surface damage is shown in Figure 2.

The minimum distance at which the leaves did not appear damaged was 3 cm, and this distance was adopted for further experiments.

In the initial set up step, lettuce leaves were processed at different time conditions (from 0 to 30 s). Based on the results achieved, the treatment time of 15 s was chosen for the shelf life evaluation. In both steps of the study, untreated leaves were used as control. For every leaf (treated and untreated), a specific area (upper right quadrant) was selected in order to minimize the variability associated with leaf anatomy. Following the treatments, the plasma-treated zone (i.e., 2 cm^2^) was separated from the untreated one by coring. Analogously, a 2 cm^2^ area was cut from each untreated sample in the selected area. All samples were packed in polypropylene food containers and stored in a refrigerator at 4 °C. Treated and untreated samples (in triplicate) were analysed in parallel immediately after the treatment (day 0) and during the shelf life period (days 1, 2, 5, 7, and 9) for chemical, physical, and microbiological parameters.

### 2.4. Lettuce Characterization

#### 2.4.1. Tissue Electrolyte Leakage and pH

For electrolyte leakage (EL) analysis, each sample consisted of three discs placed in a sterile Falcon tube containing distilled water (20 mL). Samples were incubated for 2 h at room temperature under continuous shaking. The electrical conductivity of the solution was recorded using a laboratory conductivity meter both before and after a freeze–thaw cycle and the level of leakage was expressed as the percentage difference between the two measurements.

The pH was measured after the freeze–thaw cycle using a pH-meter (pH-meter GLP 21+, Crison Instruments, Barcelona, Spain).

#### 2.4.2. Dry Matter Content

The tissue dry matter content of leaves was determined by heating samples (three discs of leaves) at 105 °C for about 8 h in an oven (Sanyo OMT Oven, Loughborough, UK), until reaching the constant weight. The samples, which were introduced in calibrated crucibles, were weighed both immediately before and after heat treatment. The dry matter content was expressed as a percentage of the initial weight of the sample.

#### 2.4.3. Microbiological Analyses

The antimicrobial efficiency of APPJ treatment was assessed by measuring the total aerobic bacterial count of the fresh-cut leaf surface. To this purpose, leaves were homogenized in sterile 2% (*w*/*v*; pH 7.5 ± 0.1) dipotassium hydrogen phosphate solution (Sigma-Aldrich, St. Louis, MO, USA) for 2 min using a Stomacher blender (BagMixer 400, Interscience, St Nom la Bretèche, France). Ten-fold serial dilutions of each sample were prepared in quarter-strength Ringer’s solution (Scharlau Microbiology, Barcelona, Spain) and plated on a Petrifilm Aerobic Count plate (3M Minneapolis, MN, USA) according to the manufacturer’s instructions. The plates were incubated at 30 °C for 72 h. Each experiment was performed in triplicate. The results were expressed as log_10_ CFU per gram of product (log_10_ CFU/g, mean values ± standard error).

### 2.5. Statistical Analysis

All data were subjected to the analysis of variance (ANOVA) followed by Sidak’s multiple comparisons test with *p*-value less than 0.05 (*p* < 0.05). Statistics were performed using GraphPad Prism version 6 for Windows [16].

## 3. Results and Discussion

### 3.1. Preliminary Set Up

#### 3.1.1. Inactivation of Autochthonous Microbiota

In order to investigate the antimicrobial efficiency of the APPJ treatments on lettuce, the surface cell concentration related to the natural bacterial community of the leaves, coming directly from the field, without any washing, was analysed. This approach gives a general and more representative picture of the microbial load to which consumers are potentially exposed at the point of sale or the time of consumption. Additionally, the increase in total mesophilic bacteria is universally used as an indicator of quality decay for this kind of product [17].

**Figure 3:**
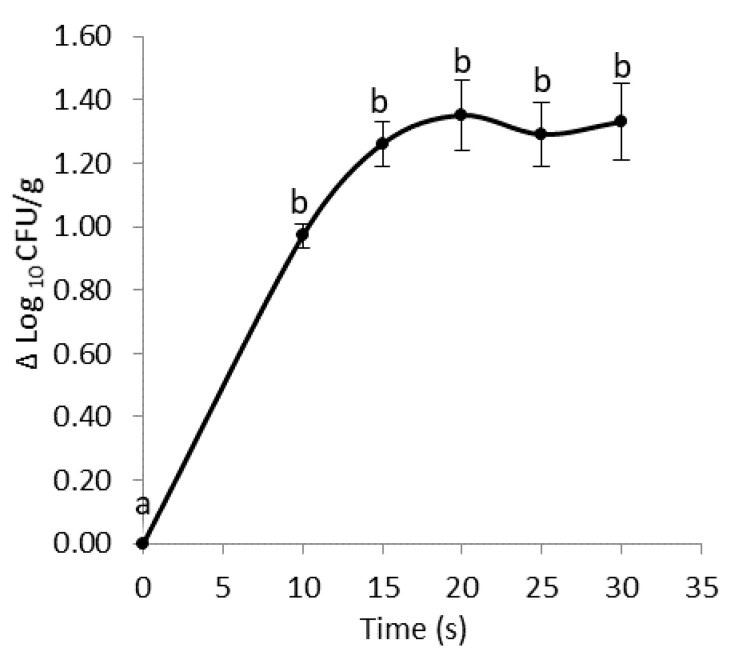
Logarithmic reduction of the total microbial count of fresh lettuce leaves after air atmospheric pressure plasma jet (APPJ) treatment in function of the treatment time (mean values ± standard error; CFU = colony forming unit). Different letters indicate statistically significant differences between time points, according to the analysis of variance (ANOVA) followed by Sidak post-hoc test (*p* < 0.05; *n* = 3).

Optimization of treatment variables for the improvement of plasma performance as an antimicrobial technology is mandatory [18]. In particular, the experimental set up had the primary purpose of identifying a minimum period of time, which ensures a satisfactory microbiological reduction, with no or minimal damage to salad quality. The duration of treatment is the main process parameter of interest in this study. Thus, the reduction of the total microbial count of green lettuce was first studied as a function of the duration of the APPJ treatment (Figure 3).

The total count of aerobic mesophilic bacteria of non-treated fresh samples was 6.98 ± 3.14 log_10_ CFU/g, a level higher than that found by other authors in different types of unwashed lettuce (3.00–5.00 log_10_ CFU/g) [6,19,20]. Previous studies highlighted the influence of the initial microbial concentration in the product on the efficacy of bacterial inactivation. In fact, when a high cell concentration occurs, microorganisms are associated into multiple layers or clumps, which protect them against the antibacterial agents (e.g., plasma radiation). On the contrary, when an initial cell number corresponding to 10^5^ CFU/mL is present, bacteria are in the form of a monolayer, thus being directly exposed to the active molecules and enhancing the antimicrobial activity [21]. The cell concentration quickly decreased during the first 10 s of treatment, resulting in a statistically significant reduction of about one logarithmic cycle in the microbial population, as illustrated in Figure 3. A further 5 s exposure determined an additional, but not significant decrease in microbial load. The APPJ treatment from 25 to 30 s did not increase the antimicrobial effect. The obtained results agree with other studies reported in the literature, describing a double-slope or biphasic plasma-inactivation kinetics [22,23,24]. The process is characterized by an initial quick reduction phase followed by a second, slower inactivation phase. The first step may be due to the plasma attack on superficial bacterial layers, present as multilayer biofilms. The second step shows a decontamination rate slowdown, which is probably due to plasma penetration through the previously treated cell layers, before reaching the deeper biofilm’s layer. The evolution from a direct to an indirect (with penetration) plasma-attack leads to a progressive slower and less efficient decontamination. The polymeric matrix surrounding the cells, which is made of the previously formed cell debris, provides protection by a shielding effect.

#### 3.1.2. Effect on the Physiological Properties of Lettuce

**Figure 4:**
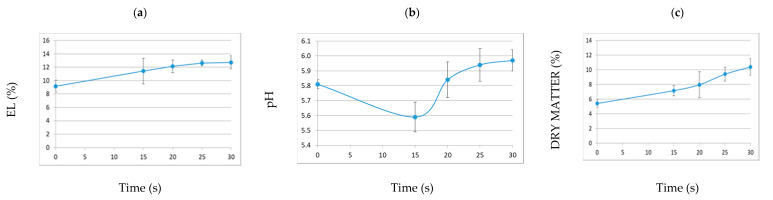
Plasma-induced changes in physicochemical parameters (electrolyte leakage (EL) (**a**), pH (**b**), and dry matter content (**c**)) of lettuce leaves as a function of the plasma treatment time (mean values ± standard error). There were no significant differences between time points, according to the ANOVA followed by Sidak post-hoc test (*p* < 0.05; *n* = 3).

Figure 4 shows the plasma-induced changes in physicochemical parameters of lettuce leaves as a function of the plasma treatment time.

EL describes the degree of plant cellular system perturbation in terms of release of solutes, thus being generally considered as a symptom of cell membrane injury during processing or handling of fresh-cut produce [25]. The EL values detected for untreated samples were probably due to the mechanical stress induced by placing samples on the support in the holder cage. Then, they increased after treatment, exhibiting an almost linear trend, with no significant difference between time points. The obtained EL values were higher than those reported for sanitizers and surfactants used for improving the microbial status of fresh lettuce [26,27], but similar or lower to those found by Paillart, Otma, and Woltering [28] for mild heat-shock treatments. No significant pH variations were observed among samples (Figure 4), and the pH range (5.59–5.97) was satisfactory for the retention of lettuce quality [29]. The dry matter content increased linearly with the duration of plasma exposure (Figure 4), with no significantly different value between treatments. The hydration status is correlated with the turgor state of the plant tissue, and it is inevitably influenced by cutting. A moisture loss inferior to 5%, as in this case (1.9%), is considered not to significantly affect the tissue hardness, and hence is acceptable [30].

### 3.2. Shelf Life Evaluation

#### 3.2.1. Changes in Microbiological Quality

**Figure 5:**
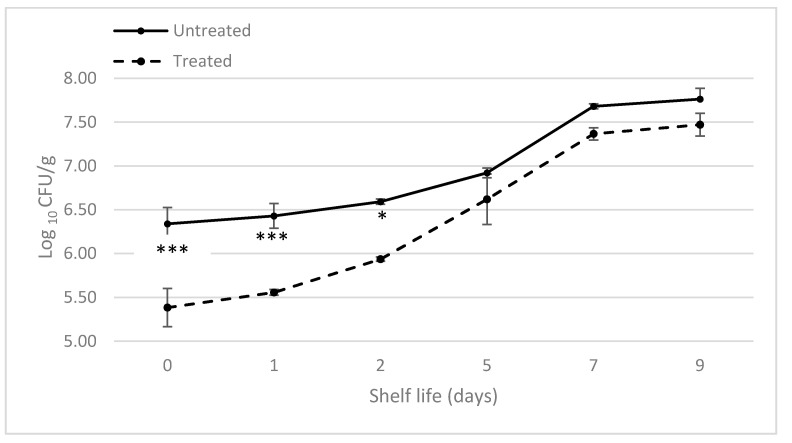
Total aerobic count of untreated and treated fresh lettuce leaves during shelf life (mean values ± standard error; CFU = colony forming unit). At each time point, asterisks indicate statistically significant differences between treatments (NT vs. T), according to the ANOVA followed by Sidak post-hoc test (*p* < 0.05; *n* = 3).

Considering the obtained results, we decided to fix the treatment time at 15 s for the antimicrobial effect evaluation during the shelf life period, a shorter treatment duration than other cold plasma configurations [31,32] applied for microbial decontamination of leafy vegetables. The choice of a 9-day shelf life study was made in order to reflect the reality, as the commercial shelf life of ready-to-eat fresh cut salads is normally indicated to be approximately equal to 7–10 days under refrigeration at ≤5 °C [33]. Figure 5 shows the total natural surface microbial amount over a 9-day period after APPJ treatment.

The application of APPJ treatment resulted in an immediate bacterial reduction. The two sample classes showed a significant initial difference (1 log cycle), which represents the logarithmic reduction achieved by the application of APPJ treatment. This value fits with the results obtained in the first research phase, and is consistent with the literature reported data [34]. Both sample classes showed a progressive increase in the total microbial concentration that could be mostly constituted by psychotropic bacteria. Actually, most of the mesophilic bacteria are able to multiply at refrigeration temperatures [35]. Unlike what Min et al. [36] reported, plasma-treated samples exhibited a higher microbial growth rate, leading to a convergence of microbiological results (0.30 log reduction) for a shelf life of more than 5 days. This is probably due to plasma-induced vegetable tissue damage, with consequent increased availability of nutrients for bacteria, which survived the plasma exposure. Unlike untreated lettuce leaves, the treated ones anyway maintained an acceptable microbiological quality throughout the shelf life period considered, as their total microbial load always was within the limit (7.70 log_10_ CFU/g) established for minimally processed fruits and vegetables in different European countries [36,37]. Plasma-treated samples were noticeably less contaminated than the non-treated ones in the first 3–4 days, causing an increase of the product’s shelf life. Despite the conceivable indigenous microbial diversity on lettuce leaves, the plasma treatment was able to retard the growth of bacteria during the early phases of refrigerated storage. Recently, Giannoglou et al. [38] obtained similar results on ready-to-eat, fresh cut, leafy rocket salad by applying a cold atmospheric plasma generated via a surface DBD. The plasma processing was able to double salad shelf life (5 days for treated salad vs. 2.5 days for control, at 2 °C), reducing the total viable count by a logarithm, without affecting its quality indices (pH, colour, and texture). In another study focused on the efficiency of an atmospheric cold plasma treatment generated by means of a DBD in the decontamination of radicchio leaves, significant reductions of intentionally inoculated *Escherichia coli* O157:H7 (1.35 log MPN/cm^2^ for 15-min treatment) and *Listeria monocytogenes* (~2 log CFU/cm^2^ for 30-min treatment) were found, with no significant changes in terms of antioxidant activity of the radicchio leaves and detrimental effects on sensory attributes during storage [32]. It is worth noting that both studies required long processing (several minutes instead of seconds).

#### 3.2.2. Changes in Quality Attributes

**Figure 6:**
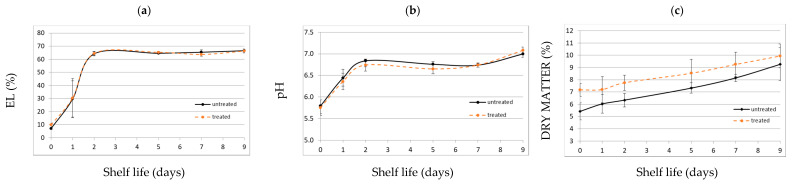
Evolution of EL (**a**), pH (**b**), and dry matter content (**c**) for untreated and treated samples over shelf life (mean values ± standard error). There were no significant differences between time points, according to the ANOVA followed by Sidak post-hoc test (*p* < 0.05; *n* = 3).

Figure 6 shows the evolution of EL (a), pH (b), and dry matter content (c) for untreated and treated samples over 9-day refrigerated storage. No significant difference emerged among samples at different storage times.

Initially, EL values were different in the two groups of samples, but the two trends converged after a day of storage. Both sample classes reached the maximum EL value at day 2, and trends became constant afterward. This suggests an enzymatic inhibition by plasma treatment, with a slowing down of the enzymatic degradation rate and subsequent convergence of the two trends, despite initial cell membrane damage, due to oxidative stress by plasma reactive species. As already noted, the EL could be stimulated by the mechanical stress induced by placing samples on the support. The treated and untreated samples showed similar pH values during shelf life, with a tendency to neutral pH values. The pH values at the beginning of storage were in agreement with the results obtained in the previous set up, confirming that the APPJ treatment (15 s) did not affect the pH of fresh cut salad. This parameter can consequently be used as an index to describe the natural aging physiological process in a food matrix. Both EL and pH trends showed an early exponential phase in the first two days of shelf life. The effects (positive or negative) induced by plasma treatment will be more relevant from a commercial perspective during this time. The evaluation of the dry matter content in both treated and untreated samples over time was carried out to determine the impact of the initial plasma-induced water loss, observed in the previous phase of the research, on the naturally occurring dehydration during storage. Actually, a common event accompanying senescence is enhanced membrane permeability owing to senescence itself and/or chilling of sensitive tissues, and expressed as increasing leakage of ions [39]. The dry matter content of both sample classes increased linearly with shelf life, but the dehydration rate was slower in the treated samples, even if they showed a lower initial moisture content. Despite the slight difference in increase rate, the two profiles tended to converge at the end of shelf life. This preliminary water loss could be related to the air jet action on the natural surface moisture layer, followed by the progressive water migration from inside to the superficial layers of the leaf. It could be interesting to assess the plasma-induced effects on the polymeric component, and if their combination with a stimulated dehydration positively or negatively regulates leaf aging.

## 4. Conclusions

In order to reduce the microbial spoilage to acceptable levels, we applied cold plasma technology, which was previously shown to combine the safety issue with quality preservation in fresh-cut produce, by reducing the enzymatic activity. The results described in this experimental work showed that APPJ could be a potential way of microbial inactivation of fresh food like salad, with a duration shorter than other studied plant configurations. In this way, APPJ could also provide a measure for food waste prevention, by increasing the shelf life, while guaranteeing food safety. Furthermore, it is worth emphasising that APPJ provides a cheap, clean, safe, and environmentally friendly method for efficient removal of microbial contaminants. However, an overall evaluation of the real effectiveness of cold-plasma technology, in terms of greater stability and durability of the product, will be possible only using a global shelf life index that takes into account several aspects of food quality (e.g., microbiology, physiology, texture, colour, and flavour).

## Data Availability

Data reported in this manuscript will be available upon request.
